# Uptake and release of amino acids in the fetal-placental unit in human pregnancies

**DOI:** 10.1371/journal.pone.0185760

**Published:** 2017-10-05

**Authors:** Maia Blomhoff Holm, Nasser Ezzatkhah Bastani, Ane Moe Holme, Manuela Zucknick, Thomas Jansson, Helga Refsum, Lars Mørkrid, Rune Blomhoff, Tore Henriksen, Trond Melbye Michelsen

**Affiliations:** 1 Department of Obstetrics, Division of Obstetrics and Gynecology, Oslo University Hospital, Oslo, Norway; 2 Institute of Clinical Medicine, University of Oslo, Oslo, Norway; 3 Department of Nutrition, Institute of Basic Medical Sciences, University of Oslo, Oslo, Norway; 4 Oslo Centre for Biostatistics and Epidemiology, Department of Biostatistics, Institute of Basic Medical Sciences, University of Oslo, Oslo, Norway; 5 Division of Reproductive Sciences, Department of OB/GYN University of Colorado Anschutz Medical Campus, Aurora, Colorado, United States of America; 6 Analytic Unit of Metabolic Diseases, Department of Medical Biochemistry, Oslo University Hospital, Oslo, Norway; 7 Department of Clinical Service, Division of Cancer Medicine, Oslo University Hospital, Oslo, Norway; 8 Norwegian Advisory Unit on Women’s Health, Oslo University Hospital, Oslo, Norway; Johns Hopkins University, UNITED STATES

## Abstract

**Objectives:**

The current concepts of human fetal-placental amino acid exchange and metabolism are mainly based on animal-, *in vitro-* and *ex vivo* models. We aimed to determine and assess the paired relationships between concentrations and arteriovenous differences of 19 amino acids on the maternal and fetal sides of the human placenta in a large study sample.

**Methods:**

This cross-sectional *in vivo* study included 179 healthy women with uncomplicated term pregnancies. During planned cesarean section, we sampled blood from incoming and outgoing vessels on the maternal (radial artery and uterine vein) and fetal (umbilical vein and artery) sides of the placenta. Amino acid concentrations were measured by liquid chromatography—tandem mass spectrometry. We calculated paired arteriovenous differences and performed Wilcoxon signed-rank tests and Spearman’s correlations.

**Results:**

In the umbilical circulation, we observed a positive venoarterial difference (fetal uptake) for 14 amino acids and a negative venoarterial difference (fetal release) for glutamic acid (p<0.001). In the maternal circulation, we observed a positive arteriovenous difference (uteroplacental uptake) for leucine (p = 0.005), isoleucine (p = 0.01), glutamic acid (p<0.001) and arginine (p = 0.04) and a negative arteriovenous difference (uteroplacental release) for tyrosine (p = 0.002), glycine (p = 0.01) and glutamine (p = 0.02). The concentrations in the maternal artery and umbilical vein were correlated for all amino acids except tryptophan, but we observed no correlations between the uteroplacental uptake and the fetal uptake or the umbilical vein concentration. Two amino acids showed a correlation between the maternal artery concentration and the fetal uptake.

**Conclusions:**

Our human *in vivo* study expands the current insight into fetal-placental amino acid exchange, and discloses some differences from what has been previously described in animals. Our findings are consistent with the concept that the fetal supply of amino acids in the human is the result of a dynamic interplay between fetal and placental amino acid metabolism and interconversions.

## Introduction

The intrauterine environment in which a fetus develops has a major impact on the immediate and future health of the newborn child. Inadequate nutrition during fetal life may cause intrauterine developmental disturbances and increase the risk of adult cardiovascular disease, diabetes, and certain cancers [1,2]. The placenta is the organ that constitutes the anatomical and metabolic interface between the maternal and fetal circulations and thus, to a large extent, governs the environment in which the fetus develops.

Normal fetal development depends on a continuous supply of amino acids (AA), and reduced AA concentrations in the fetal circulation are associated with compromised growth *in utero* [3–5]. The supply of AAs to the fetus is affected by the maternal AA supply to the placenta, placental blood flow, the capacity of placental AA transport systems, and placental and fetal AA metabolism [6]. Placental AA transport is active, i.e., energy dependent, and occurs against a concentration gradient. This transport is mediated by over 20 distinct AA transport systems, which are present in the microvillous and basal plasma membranes of the placental syncytiotrophoblast [7,8]. These transport systems have overlapping specificity and are regulated by nutrients, hormones, adipokines and cytokines [9]. The placenta itself is also known to synthesize, degrade and transaminate AAs. Inter-organ cycling and interconversions between the fetal liver and the placenta have been demonstrated for some AAs [10].

The majority of previous studies of placental amino acid exchange and metabolism have been performed in a range of animal models (e.g., sheep, rat, guinea pig, baboon, rhesus monkey, pig and horse) [10]. Large interspecies differences exist in placental structure and functions [11], and extrapolation of results from animals to humans must be done with caution. Human studies are largely based on *in vitro* and *ex vivo* models and *in vivo* AA infusions with stable isotopes [10,12]. A few, smaller human *in vivo* studies have explored placental and fetal AA uptake and release under normal physiological conditions by measuring plasma concentrations and arteriovenous concentration differences in the incoming and outgoing vessels of the uteroplacental unit in the maternal circulation and/or of the fetus in the umbilical circulation [4,13–20]. These studies have demonstrated that AA concentrations and arteriovenous concentration differences vary greatly between individuals. It is therefore questionable if the number of individuals in these studies were sufficient to draw reliable conclusions. Further, no human *in vivo* studies have, to our knowledge, assessed the paired relationships between AA concentration and arteriovenous concentration differences on both the maternal and fetal sides of the placenta in order to examine the interplay between the mother, the placenta and the fetus *in vivo*.

On this background, we aimed to determine the concentrations and paired arteriovenous differences of 19 circulating proteogenic AAs on both the maternal and fetal sides of the placenta in a large study sample *in vivo*. We further wished to assess the relationships between AA concentrations and arteriovenous concentration differences on both the maternal and fetal sides of the placenta in the same mother-fetus pairs. These data allowed us to perform an *in vivo* evaluation of some of the current concepts of fetal-placental exchange and metabolism of AAs in humans.

## Materials and methods

### Ethics approval and consent to participate

All participants signed a written informed consent. The study was approved by the data protection officials at Oslo University Hospital and the Regional Committee for Medical and Health Research Ethics, Southern Norway 2419/2011.

### Design and study population

We performed a cross-sectional *in vivo* study of 179 women scheduled for planned cesarean sections at Oslo University Hospital, Rikshospitalet between October 2012 and June 2015, and their infants. We invited healthy, non-smoking women with uncomplicated singleton pregnancies to participate. Provided that the women were healthy (absence of diagnosed disease) we intentionally included women with the whole range of BMIs and infant birthweights. In a general healthy population exclusion of BMI and birth weight categories will artificially omit information inherent in the metabolism across the weight ranges. Exclusion criteria were significant pre-existing comorbidity, medication (other than levothyroxine and occasional use of antiallergics, antiemetics, antibiotics, and antacids), pregnancy complications and onset of labor prior to scheduled cesarean section. We did not exclude women with gestational diabetes not requiring insulin or other medication based on the rationale that there is no clear metabolic distinction between this group and those defined not to have diabetes.

### Data collection

The procedure for blood sampling has been previously described in detail [21,22]. Briefly, we obtained fasting (median [Q1, Q3] duration: 10 [9, 11] hours) maternal blood samples from the radial artery and the uterine vein on the anterolateral surface of the uterus during planned cesarean section. Fetal blood was sampled from the umbilical artery and vein immediately after delivery of the infant, before delivery of the placenta. The blood was transferred to EDTA-containing vacutainers and stored on ice. Whole blood was directly analyzed for hemoglobin concentration in a blood gas analyzer (maternal blood: Radiometer ABL825 Flex, umbilical blood: Radiometer ABL90 Flex). We centrifuged the blood samples at 6°C, 2500 *g* for 20 minutes and stored the plasma samples at -80°C until analysis.

### Amino acid analyses

The concentrations of plasma AAs were analyzed by liquid chromatography—tandem mass spectrometry (LC-MS/MS) using modifications of a previously described method [23]. Briefly, we added plasma samples, calibration standards, quality control samples, and blank matrix (water) (10 μL) to a 1 mL 96 well extraction plate. A deuterated internal standard mix of all AAs (10 μL) was added to all wells. We further added an ammonium solution [10 mmol/L] (10 μL) to neutralize the samples, before adding 10 μL of 100 mmol/L 1,4 dithioerythritol to reduce cystine and cysteine mixed disulfides into reduced cysteine. The solutions were gently mixed using a plate shaker at room temperature for 15 minutes, before perchloric acid [20%, 3.3 M] (10 μL) was added to precipitate the plasma proteins. Subsequently, we mixed the samples using a plate shaker and centrifuged at 4000 rpm for 15 minutes. We then added water/sodium 1-heptane sulfonate [1mol/L]/perchloric acid [20%, 3.3 mol/L] 5/3/1 v/v/v (180 μL) diluent to a shallow 96 well analysis plate. Supernatant (20 μL) from the extraction plate was spiked into the diluent in the analysis plate, and the solutions were gently mixed on a plate shaker. We centrifuged the plate at 4000 rpm for 5 minutes and submitted for LC-MS/MS.

We analyzed the extracted AAs with an Applied Biosystems 4000 Q TRAP linear MS/MS spectrometer (Foster City, CA, USA). The chromatographic separation was performed on a Phenomenex Kinetex Core Shell C18 (100 x 4.6 mm, 2.6 μm) HPLC column and temperature setting of 30°C. The mobile phases were (A) water/formic acid (100:0.05, v/v) and (B) acetonitrile /formic acid (100:0.05, v/v) at a flow rate of 0.2 mL/min. We achieved separation with a linear gradient from 100% (A) for 2 min, 40% (A) from 2 to 6 min followed by a linear gradient back to 100% (A) over 2 min. The whole run was 6.5 min and the injection volume was 25 μL. Each AA was identified by MS, corresponding to each particular internal standard and the concentration of each AA was determined from the ratio of analyte peak area/internal standard peak area against a linear multiple point calibration curve. Accuracy and the intra-day and inter-day precision of the assay were determined by analyzing two different quality control samples and calculating the mean value, standard deviation and coefficient of variation. The coefficient of variation (%) was between 6.3 and 9.9 for all AAs, except for lysine (17.2) and serine (19.9). We normalized each AA against the mean value of the quality control to adjust for day-to-day variation in the analyses.

### Calculation of uteroplacental arteriovenous differences and umbilical venoarterial differences

On the maternal side of the placenta, we assumed similar blood composition in the radial and uterine artery. The paired uteroplacental arteriovenous concentration differences were thus calculated as the difference in a given AA concentration between the radial artery and the uterine vein. On the fetal side of the placenta, the paired umbilical venoarterial differences were calculated as the difference in a given AA concentration between the umbilical vein and the umbilical artery. A positive concentration difference was defined as uptake, i.e., a uteroplacental uptake from the maternal circulation or a fetal uptake from the umbilical circulation (the latter also corresponded to a placental release to the umbilical circulation). A negative concentration difference was defined as release, i.e., a uteroplacental release to the maternal circulation or a fetal release to the umbilical circulation (the latter also corresponded to a placental uptake from the umbilical circulation).

### Adjustment for changes in plasma water content

A net transfer of water between the maternal, placental and fetal compartments may affect the calculated arteriovenous concentration differences. The ratio of Hb concentrations between the outgoing and incoming vessels may be used as to assess the net passage of water across the placenta. In representative sub cohorts (maternal circulation n = 40, umbilical circulation n = 30) we studied the ratios of hemoglobin (Hb) concentrations in incoming and outgoing vessels on both sides of the placenta during maternal fasting. We observed a median [Q1, Q3] uterine vein vs. radial artery Hb ratio of 1.017 [1.002, 1.029] (range 0.98–1.06), consistent with a uteroplacental uptake of water from the maternal circulation. Further, we observed a median [Q1, Q3] umbilical artery vs vein Hb ratio of 1.048 [1,01, 1.077] (range 0.94–1.16), consistent with a fetal uptake from the umbilical circulation.

In order to take into account the net passage of water across the placenta, we divided the different AA concentrations ([X] for AA X) in the uterine vein and umbilical artery in all the mother-fetus pairs in the study with the median maternal and fetal Hb ratio, respectively, to adjust the arteriovenous and venoarterial concentration differences:

$$Uteroplacental\;arteriovenous\;difference\;\left[X\right]={\left[X\right]}_{radial\;artery}-\left(\frac{{\left[X\right]}_{uterine\;vein}}{1.017}\right)$$

and

$$Umbilical\;venoarterial\;difference\;\left[X\right]={\left[X\right]}_{umbilical\;vein}-\left(\frac{{\left[X\right]}_{umbilical\;artery}}{1.048}\right)$$

### Statistical analyses

Clinical data are presented as mean values with standard deviations or numbers with percentages, as appropriate. BMI and AA concentrations are presented as medians with quartiles because their distributions are often skewed. All AA variables were treated as continuous data. We performed paired comparisons between AA concentrations in the different vessels with Wilcoxon sign rank tests and correlation analysis with Spearman’s rank correlation coefficient. All statistical tests were performed as two-sided tests. P-values were adjusted for multiple testing by controlling the false discovery rate (FDR) at the level of 0.1 with the method of Benjamini-Hochberg [24]. In cases of missing data due to incomplete sampling, the statistical analyses were performed with the available data. We performed all analyses using Statistical Package for the Social Sciences, Version 21.0 (SPSS Inc., Hong Kong).

## Results

### Clinical characteristics

Table 1 shows the clinical characteristics of the participating women and their infants. The women had a mean age of 35.4 years, and 86.6% were highly educated. They were generally lean, with median BMI of 22.3 kg/m^2^ before pregnancy and mean gestational weight gain of 15 kg. The range in these two parameters were, however, quite large, reflecting the population we wished to study. The infants were born at term with a wide range of birth weights and placental weights.

**Table 1:** Clinical characteristics of the participating women and infants.

	n	%	Mean (SD)/median [Q1, Q3]	Range
**Women**	179			
Age (years)			35.4 (3.8)	23–44
Para≥1	135	75.4		
BMI before pregnancy (kg/m^2^)^a^			22.3 [20.8, 25.4]	17.0–47.6
BMI at delivery (kg/m^2^)^a^			28.2 [26.1, 31.2]	21.4–48.4
Gestational weight gain (kg)			15.0 (4.8)	-1.2–31.3
Married or partnership	174	97.2		
Higher education (>15 years)	155	86.6		
Smoking during pregnancy^b^	0	0		
Gestational diabetes^c^	4	2.2		
**Infants**	179			
Gestational age (weeks)			39.3 (0.6)	37.1–42.0
Birthweight (g)			3546 (443)	2297–4955
Length (cm)			50.7 (1.7)	46–55
Placenta weight (g)^d^			617.2 (133.4)	310–1115
Sex (boys)	99	55.3		
Apgar score < 7 after 5 min	0	0		

^a^Value presented as median [Q1, Q3] due to skewed distribution

^b^Seven women stopped smoking when pregnancy was confirmed in first trimester.

^c^Not insulin treated. Defined based on WHO criteria: Fasting plasma glucose of 5.1–6.9 mmol/L or plasma glucose of 8.5–11.0 mmol/L 2 hours after an oral glucose tolerance test of 75 g glucose.

^d^Untrimmed, without blood clots.

### Amino acid concentrations

Table 2 shows the median and quartiles of the concentrations of 19 AAs in the radial artery, uterine vein, umbilical vein and umbilical artery. The median concentration of all but three AAs was significantly higher in the fetal circulation (umbilical vein) compared with the maternal circulation (radial artery) (aspartate p = 0.002, others p<0.001) when assessed Wilcoxon sign rank tests. Cysteine (p<0.001) and glutamic acid (p<0.001) concentrations were significantly higher in the maternal circulation, while methionine (p = 0.39) showed no statistical difference between the maternal and fetal circulations. Lysine showed the largest concentration difference, with a fetal to maternal ratio of 2.9. Glycine and tryptophan were also more than twice as high in the umbilical circulation compared with the maternal circulation. Glutamine was present in the highest concentrations in all four vessels. Its concentration was more than three times higher than alanine, which had the second highest concentration in the maternal circulation, and more than twice the concentration of lysine, which was the second most abundant AA in the umbilical circulation. Methionine showed the lowest concentrations in both the maternal and umbilical circulation. Notably, we observed large variations in the AAs concentrations among the different individuals.

**Table 2:** Amino acid concentrations on the maternal and fetal sides of the placenta.

Amino acid	Radial artery μmol/L	Uterine vein μmol/L	Umbilical vein μmol/L	Umbilical artery μmol/L
N	168	177	177	162
**Essential**				
Histidine	92.1 [82.0, 101]	91.7 [79.8, 104]	120 [106, 145]	117 [100, 141]
Isoleucine	48.8 [41.9, 57.6]	48.1 [39.4, 55.8]	69.2 [61.1, 84.0]	65.0 [54.0, 74.2]
Leucine	108 [81.8, 129]	103 [80.1, 127]	157 [121, 192]	139 [113, 171]
Lysine	143 [87.9, 245]	149 [85.4, 243]	415 [209, 597]	359 [230, 541]
Methionine	13.2 [10.1, 16.5]	12.7 [10.2, 16.7]	13.8 [10.1, 17.9]	13.5 [10.8, 17.7]
Phenylalanine	49.2 [41.9, 54.8]	49.1 [43.6, 58.3]	76.3 [66.7, 90.6]	72.8 [63.5, 86.0]
Threonine	162 [127, 216]	161 [128, 208]	238 [192, 319]	237 [190, 317]
Tryptophan	40.1 [31.7, 48.8]	41.1 [32.7, 50.5]	88.3 [76.1, 105]	83.0 [69.6, 98.8]
Valine	157 [133, 187]	158 [134, 196]	259 [222, 308]	243 [208, 281]
**Non-essential**				
Alanine	243 [209, 276]	255 [2, 294]	316 [283, 367]	275 [241, 311]
Arginine	57.2 [47.4, 68.2]	56.6 [47.6, 66.8]	111 [96.7, 126]	106 [90.9, 122]
Aspartate	39.6 [29.0, 52.7]	41.5 [30.3, 56.7]	44.6 [32.3, 59.9]	43.2 [32.5, 60.2]
Cysteine	221 [195, 248]	226 [195, 249]	202 [184, 225]	199 [179, 217]
Glutamic acid	62.4 [51.7, 79.4]	51.5 [36.3, 65.3]	39.3 [24.6, 53.5]	45.0 [33.3, 60.9]
Glutamine	863 [774, 978]	891 [808, 1000]	1130 [1035, 1270]	1140 [1040, 1280]
Glycine	111 [74.8, 150]	114 [75.3, 183]	231 [190, 283]	239 [200, 290]
Proline	120 [101, 145]	125 [103, 157]	169 [144, 196]	163 [139, 187]
Serine	115 [87.0, 143]	113 [83.6, 139]	148 [116, 185]	162 [135, 204]
Tyrosine	33.6 [27.4, 42.0]	37.6 [31.4, 44.9]	63.6 [54.1, 78.3]	64.9 [55.6, 74.2]

Concentrations are presented as medians [Q1, Q3]

### Uteroplacental arteriovenous and umbilical venoarterial amino acid differences

**Fig 1:**
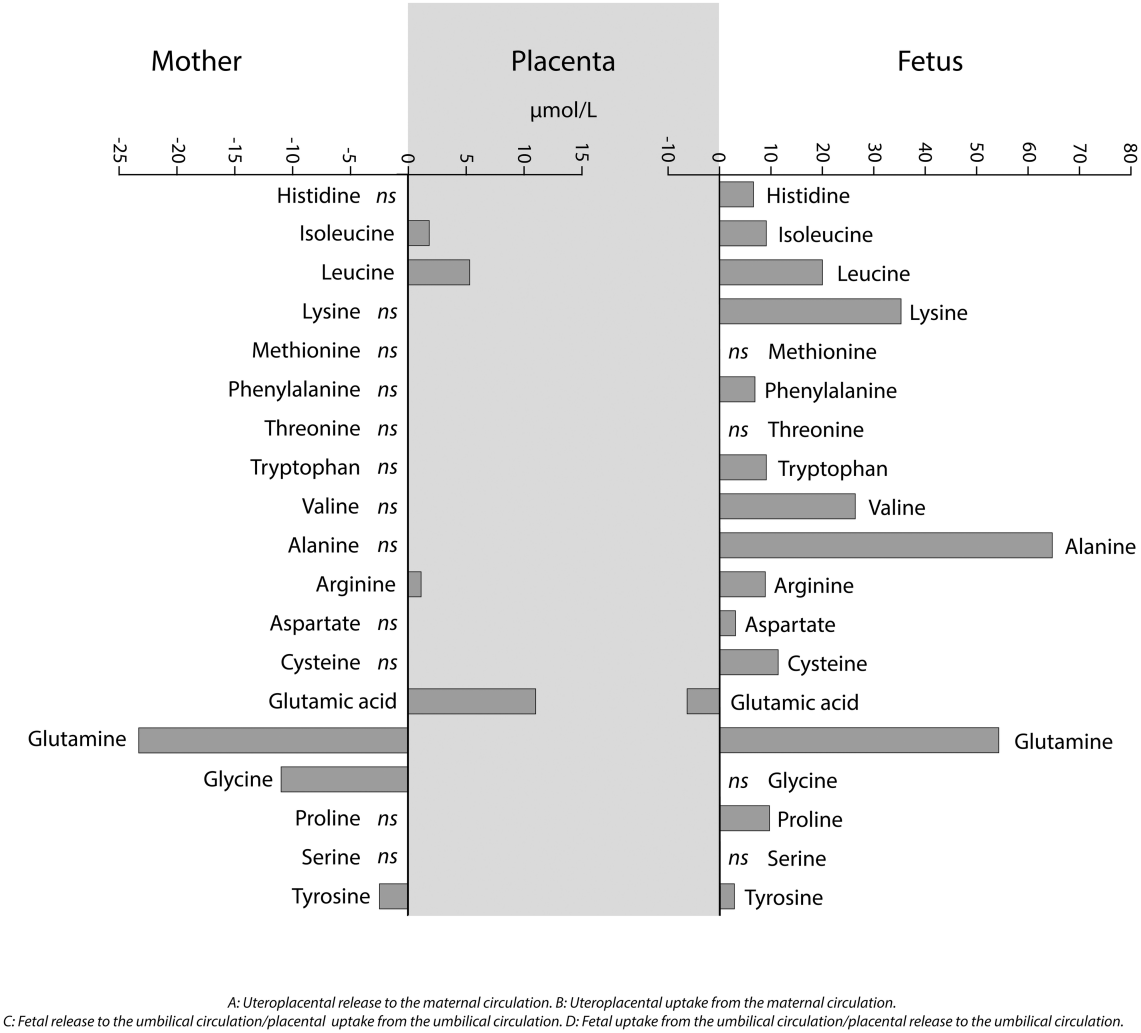
Summary of uteroplacental arteriovenous and umbilical venoarterial amino acid concentration differences adjusted for transfer of water across the placenta. Medians of significant concentration differences after adjustment for multiple testing by controlling the false discovery rate (FDR) according to the method of Benjamini and Hochberg are shown.

Table 3 shows the median and quartiles of the paired AA concentration differences on the maternal and fetal sides of the placenta with p-values. Both crude concentration differences and concentration differences adjusted for transfer of water across the placenta are shown. We assessed the paired concentration differences on the maternal and the fetal sides with Wilcoxon sign rank tests. These data are summarized in Fig 1.

**Table 3:** Crude and adjusted uteroplacental arteriovenous and umbilical venoarterial concentration differences with p-values.

Amino acid	Uteroplacental arteriovenous difference μmol/L				Umbilical venoarterial difference μmol/L			
n	166				160			
Essential	Crude	p-value	Adjusted for transfer of water across the placenta	p-value	Crude	p-value	Adjusted for transfer of water across the placenta	p-value
Histidine	-0.1 [-6.3, 6.2]	0.77	1.2 [-5.1, 7.5]	0.15	**1.7 [-7.0, 15.5]**	0.03	**6.6 [-0.7, 20.0]**	<0.001
Isoleucine	1.1 [-5.6, 6.8]	0.15	**1.8 [-4.5, 7.4]**	0.01	**6.4 [-4.3, 17.7]**	<0.001	**9.0[-0.4, 21.5]**	<0.001
Leucine	1.7 [-7.8, 11.8]	0.12	**5.3 [-6.0, 13.8]**	0.005	**12.6 [-3.6, 31.8]**	<0.001	**20.0 [2.1, 38.5]**	<0.001
Lysine	6.3 [-54.3, 53.5]	0.67	8.7 [-52.1, 55.4]	0.46	25.5 [-77.5, 159]	0.06	**35.3 [-55.9, 182]**	0.004
Methionine	0.0 [-2.9, 3.2]	0.68	0.2 [-2.6, 3.4]	0.35	-0.4 [-3.1, 2.5]	0.49	0.2 [-2.5, 3.1]	0.30
Phenylalanine	-1.4 [-6.8, 5.2]	0.21	-0.5 [-5.8, 5.8]	0.86	**3.6 [-6.0, 13.2]**	0.02	**6.9 [-2.2, 16.1]**	<0.001
Threonine	-2.5 [-29.9, 36.0]	0.99	0.6 [-27.3, 38.7]	0.52	0.0 [-39.8, 45.5]	0.89	14.5 [-27.8, 55.6]	0.07
Tryptophan	-0.3 [-7.6, 6.0]	0.49	0.4 [-7.1, 6.5]	0.90	**4.9 [-8.9, 20.0]**	0.001	**9.1 [-4.9, 23.1]**	<0.001
Valine	**-8.5 [-17.1, 7.0]**	<0.001	-5.7 [-13.6, 9.7]	0.05	**14.4 [-8.5, 41.3]**	<0.001	**26.4 [2.3, 51.9]**	<0.001
**Non-essential**								
Alanine	**-6.0 [-35.0, 21.3]**	0.02	-1.1 [-30.2, 25.6]	0.26	**52.0 [18.0, 79.8]**	<0.001	**64.7 [29.4, 92.1]**	<0.001
Arginine	0.3 [-4.3, 5.2]	0.60	**1.1 [-3.3, 6.0]**	0.04	**4.0 [-6.9, 11.7]**	0.006	**8.9 [-1.7, 16.0]**	<0.001
Aspartate	-1.0 [-11.5, 9.3]	0.47	-0.3 [-10.7, 10.2]	0.82	1.2 [-8.8, 10.5]	0.27	**3.1 [-6.0, 12.1]**	0.007
Cysteine	-1.1 [-28.0, 23.1]	0.53	2.5 [-23.7, 26.9]	0.47	2.1 [-18.9, 26.4]	0.14	**11.4 [-8.4, 34.4]**	<0.001
Glutamic acid	**10.3 [1.3, 21.9]**	<0.001	**11.0 [2.6, 22.8]**	<0.001	**-8.7 [-16.6, 0.2]**	<0.001	**-6.3 [-14.4, 3.3]**	<0.001
Glutamine	**-39.0 [-97.0, 31.3]**	<0.001	**-23.3 [-80.1, 43.9]**	0.02	1.5 [-60.0, 70.0]	0.74	**54.3 [-12.1, 118]**	<0.001
Glycine	**-13.0 [-47.2, 25.3]**	0.004	**-11.0 [-43.5, 27.4]**	0.01	-2.0 [-65.0, 42.3]	0.21	9.8 [-53.8, 54.2]	0.73
Proline	**-3.5 [-20.0, 11.1]**	0.02	-1.2 [-17.5, 12.8]	0.21	3.0 [-14.0, 25.0]	0.07	**9.7 [-5.6, 31.6]**	<0.001
Serine	0.6 [-27.2, 24.1]	0.96	2.3 [-25.0, 25.7]	0.63	**-10.5 [-47.5, 26.8]**	0.03	-2.1 [-38.5, 33.1]	0.45
Tyrosine	**-3.2 [-8.6, 3.5]**	<0.001	**-2.5 [-8.0, 4.0]**	0.002	0.2 [-7.6, 8.3]	0.93	**2.9 [-4.2, 11.1]**	0.002

Median concentration differences are presented crude and adjusted for transfer of water across the placenta [Q1, Q3]. A positive value indicate uptake by the placenta on the maternal side and by the fetus on the umbilical side. Significant differences after adjustment for multiple testing by controlling the false discovery rate (FDR) according to the method of Benjamini and Hochberg are marked in bold. Note that the FDR adjusted p-values for significant results are reported in the text.

On the fetal side of the placenta, we observed a significant crude positive umbilical venoarterial difference for 8 AAs. When transfer of water across the placenta was taken into account, we observed a positive venoarterial difference for 14 AAs. These AAs included all the essential AAs, except methionine and threonine. All of the concentration differences remained significant after adjustment for multiple testing (p<0.001 for all except lycine (p = 0.005), aspartate (p = 0.009) and tyrosine (p = 0.003)). Alanine showed the largest positive concentration difference in the umbilical circulation, both absolutely and the percent of its plasma concentration in the umbilical vein (16%), followed by isoleucine (9%) and leucine (8%). Glutamic acid (p<0.001) and serine (p = 0.03) were the only AAs that showed a significant negative umbilical venoarterial difference, however only the concentration difference for glutamic acid was significant when we adjusted for transfer of water across the placenta and multiple testing (p<0.001). Glutamic acid also showed the largest venoarterial difference overall in the per cent of the concentration in the umbilical vein (22%).

On the maternal side of the placenta, the uteroplacental arteriovenous difference was significantly positive for glutamic acid (p<0.001), and further for leucine (p = 0.005), isoleucine (p = 0.01), and arginine (p = 0.04) when transfer of water across the placenta was taken into account. The concentration differences remained significant after adjustment for multiple testing (glutamic acid (p<0.001), leucine (p = 0.03), isoleucine (p = 0.05) and arginine (p = 0.095)). Glutamic acid showed the largest positive concentration difference, both absolutely and in the percent of its concentration in the radial artery (17%). Six AAs showed a significant crude negative uteroplacental arteriovenous difference, but only the concentration differences for tyrosine (p = 0.002), glycine (p = 0.01) and glutamine (p = 0.02) were significant when we adjusted for the transfer of water across the placenta. The concentration differences remained significant after adjustment for multiple testing (tyrosine (p = 0.02), glycine (p = 0.05) and glutamine (p = 0.05). Glutamine showed the largest negative arteriovenous concentration difference, while glycine showed the largest concentration difference in the percent of its concentration in the radial artery (12%). Consistent with the variability in absolute AA concentrations, we observed large individual variations in the concentration differences on both the maternal and fetal sides of the placenta, with both negative and positive concentration differences for all AAs.

### Correlations

We observed significant Spearman rank correlations between the concentration in the maternal radial artery and the concentration in the umbilical vein for all the analyzed AAs except for tryptophan (r_s_ = 0.14, p = 0.07). Glutamic acid showed a negative correlation (r_s_ = -0.20, p = 0.01), whereas the other AAs showed a positive correlation (r_s_ = 0.24–0.79, p <0.001 for all except methionine; p = 0.002) (S1 Table). The p-values remained the same after adjustment for multiple testing for all AAs. There were no significant correlations between the uteroplacental arteriovenous differences and the AA concentrations in the umbilical vein. Further, only phenylalanine showed a significant correlation between the uteroplacental arteriovenous difference and the umbilical venoarterial difference (r_s_ = 0.18, p = 0.04). This correlation was not significant after adjustment for placental transfer of water and multiple testing (p = 0.59). We did observe significant negative correlations between the uteroplacental arteriovenous differences of phenylalanine (r_s_ = -0.22, p = 0.006), glutamic acid (r_s_ = -0.17, p = 0.04) and glycine (r_s_ = -0.21, p = 0.009) and their concentrations in the umbilical artery. However, only the correlations for phenylalanine and glycine were significant after adjustment for multiple testing (p = 0.09 for both), and they did not remain significant after adjustment for placental transfer of water (p = 0.11 for both). The umbilical venoarterial differences of leucine (r_s_ = 0.31, p<0.001) and alanine (r_s_ = 0.27, p = 0.001) were correlated with their concentrations in the radial artery. After adjustment for multiple testing the p-values were <0.001 for leucine and 0.01 for alanine. This correlation was also observed for histidine (r_s_ = 0.19, p = 0.02), valine (r_s_ = 0.18, p = 0.03) and aspartate (r_s_ = 0.16, p = 0.05) when we adjusted for placental transfer of water, but they did not remain significant after adjustment for multiple testing (p = 0.11, p = 0.12 and p = 0.18, respectively).

## Discussion

We report the fasting concentrations and paired arteriovenous differences of 19 circulating proteogenic AAs on both the maternal and fetal sides of the placenta in a large study sample *in vivo*. We observed a significant crude uptake (μmol/L blood passed) of 8 AAs by the fetus from the umbilical circulation, and a significant fetal uptake of 14 AAs when we took into account the transfer of water across the placenta. Most of the essential AAs showed a fetal uptake. In agreement with our results, the previous smaller studies on venoarterial differences in the umbilical circulation have shown a fetal uptake of most of the essential AAs and a fetal release of glutamic acid [4,13–15,18–20]. For the remaining AAs, the reported umbilical venoarterial differences vary. On the maternal side of the placenta, the data are more conflicting. Our data showed a crude uteroplacental uptake of glutamic acid, and in addition an uptake of arginine and the essential AAs leucine and isoleucine from the maternal circulation when placental transfer of water was taken into account. We further observed a uteroplacental release of six AA to the maternal circulation, but only glutamine, glycine and tyrosine showed a uteroplacental release when we adjusted for placental transfer of water. Human infusion studies with stable isotope methodologies have demonstrated a significant placental uptake of most essential AAs from the maternal circulation, in particular leucine, isoleucine, phenylalanine and methionine [25–27]. In contrast, previous smaller human *in vivo* studies of physiological concentrations and arteriovenous differences on the maternal side of the placenta have only been able to detect a significant uteroplacental uptake of the non-essential AAs glutamic acid and aspartate, and further a release of glycine, alanine and threonine [13,16,19,20].

The current study is, to our knowledge, the first to assess paired relationships between AA concentrations on both the maternal and fetal sides of the placenta and uteroplacental arteriovenous and umbilical venoarterial concentrations differences in humans *in vivo*. We observed a correlation between the concentration in the radial artery, representing the maternal arterial supply to the placenta, and the concentration in the umbilical vein, representing the umbilical supply to the fetus, for each of the analyzed AAs except tryptophan. The observed correlation between maternal and umbilical concentrations for most AAs is in agreement with previous reports [3,4,17] and has been considered as an indication of a direct trans-placental transport of AAs from mother to fetus. However, the current study shows that the uteroplacental uptake of the different AAs per liter blood passing the placenta in the maternal circulation was neither correlated with the concentrations of the corresponding AA in the umbilical vein nor with the uptake by the fetus. Further, we observed that only the fetal uptake of leucine and alanine was significantly correlated with their maternal arterial concentrations. This limited relation between maternal AA concentrations and uteroplacental uptake on one hand and fetal uptake on the other is in line with human and animal studies of maternal AA infusions, suggesting that an increase of maternal AA concentrations does not necessarily increase the uptake of AAs in the placenta and fetus [28–30].

It is reasonable to assume that all essential and several nonessential AAs are transferred by the placenta from the maternal circulation to meet fetal demand. Our observation of higher AA concentrations in the umbilical circulation compared with the maternal circulation is, in agreement with previous reports [3,31,32], consistent with an active maternal to fetal transport across the placenta. However, the current study also shows how this transfer is complex and dynamic, and that the human placenta itself may play an important role in the governing of fetal AA supply. Uteroplacental uptake of AAs from the mother may predominantly occur in the post-prandial state, or fluctuate between uptake and release, with a net transfer to the fetus over time. The latter is consistent with the marked inter-individual differences, with uteroplacental uptake in some subjects and release into the maternal circulation in others for all AAs. At a given time and in a given maternal metabolic state, there may be a fetal uptake of AAs from the umbilical circulation without a corresponding uteroplacental uptake from the maternal circulation due to placental metabolism and interconversion of AAs. Even keto-forms of essential AAs may be taken up from the maternal and fetal circulations and subjected to amination in the placenta, thus providing essential AAs for the fetus without direct placental uptake from the maternal circulation [33].

Our data on *in vivo* placental AA transfer and exchange in women differs from several of the concepts derived from animal studies. For example, the current study shows a uteroplacental uptake of glutamic acid and a release of glutamine on the maternal side of the placenta, while previous animal studies have shown no significant uteroplacental uptake of glutamic acid and a considerable uteroplacental uptake of glutamine [10,34,35]. Our observations are consistent with *in vitro* studies showing that uptake of glutamic acid by the human placenta is converted to glutamine which is largely released to the maternal side of the placenta [36]. Further, our data are not consistent with the concept of cycling of serine and glycine between the placenta and fetal liver that has been described in animals [34,37]. We observed no placental delivery of glycine to the umbilical circulation and no significant placental uptake of serine from the maternal or umbilical circulations when we adjusted for transfer of water across the placenta. We did, however, observe a uteroplacental release of glycine to the maternal circulation. The correlation between this release and the glycine concentration in the umbilical artery shown in the current study suggests that the uteroplacental release of glycine is a result of excess glycine in the fetal-placental unit, which may be produced by the fetus itself. The observed net release of glutamine and glycine, which are two neutral non-essential AAs transported by the System L, from the uteroplacental unit into the maternal circulation may be critical for the utero-placental uptake of essential AAs in the human placenta. System L is an exchanger that uses the energy of the outwardly directed gradient of non-essential AAs, which is established by other AA transporter systems, such as System A, to drive the uptake of essential AAs into the syncytiotrophoblast against its concentration gradient [38].

It is crucial to consider the transfer of water across the placenta when studying arteriovenous concentration differences of AAs. In representative sub cohorts in the present study we observed a change in hemoglobin concentrations following the passage through the placenta, consistent with a net uteroplacental uptake of water from the maternal circulation and a net fetal uptake of water from the umbilical circulation. Consequently, without considering placental transfer of water we generally underestimate fetal uptake and overestimate fetal release of AAs in the umbilical circulation, and underestimate uteroplacental uptake and overestimate uteroplacental release in the maternal circulation. In the present study we only had access to median Hb ratios to adjust the individual AA concentration differences, and there are therefore uncertainties related to the adjusted concentration differences presented. Despite these uncertainties our findings illustrate that placental water exchange needs to be considered in studies of transfer of compounds across the placenta. There is also a need for improved methods to assess water exchange in the fetal-placental unit.

Major strengths of the current work are the *in vivo* sampling method with paired plasma samples and the LC-MS/MS methodology which is considered the gold standard for amino acid analyses [39]. This is the first time this highly sensitive method has been used to analyze plasma samples in the fetal-placental unit in the human. Another important strength is the substantial number of participants, since the fetal-placental AA concentrations and arteriovenous differences show large individual variations. We cannot exclude that there may exist small but physiologically significant concentration differences that would require an even higher number of participants to detect. The less sensitive analyzing techniques and smaller study samples without consideration of the transfer of water across the placenta make the results of the previous human *in vivo* studies of amino acid concentrations and arteriovenous differences uncertain in terms of drawing reliable conclusions. All of the women in the present study were fasting at the time of the blood sampling and were thus in a basal euglycemic state (mean glucose (SD): 4.41 (0.44) mmol/L, median insulin [Q1, Q3]: 51.7 [34.2, 77.2] pmol/L). This criterion is an important standardization of our method because a basal metabolic state normally prevails during a large proportion of the day.

A limitation of the present study is its cross-sectional design. We could reasonably only obtain blood samples at term and our data may therefore not fully represent *in vivo* placental AA transfer earlier in pregnancy. Further, our estimates of uteroplacental AA uptake and release in the maternal circulation are based on the concentration differences between the radial artery and the uterine vein. As the uterine vein drains the uterine muscular tissue in addition to the placenta, we cannot exclude a uterine contribution to our observations.

In conclusion, the current work shows how the net supply of AA to the fetus is the result of a dynamic interplay between fetal and placental AA metabolism and interconversions. Our present observations expand the current understanding of fetal-placental AA exchange in humans, and disclose some distinct differences from what has been described previously in animal models. Our *in vivo* sampling approach may add important basic knowledge to the prevailing concepts of human placental function and how it regulates the intrauterine nutritional environment under normal physiological conditions.

## Supporting information

S1 Table Spearman’s correlations (r_s_) between amino acid concentrations and arteriovenous and venoarterial concentration differences with p-values. Correlations with both crude concentration differences and concentration differences adjusted for transfer of water across the placenta are shown. Significant differences after adjustment for multiple testing by controlling the false discovery rate (FDR) according to the method of Benjamini and Hochberg are marked in bold. Note that the FDR adjusted p-values for significant results are reported in the text.
(DOCX)
