# Supplementary material for: Uptake and release of amino acids in the fetal-placental unit in human pregnancies
Source: PLoS One. 2017 Oct 5;12(10):e0185760. doi: 10.1371/journal.pone.0185760 (PMC5628923; doi:10.1371/journal.pone.0185760)
Supplement: S1 Table — Correlations with both crude concentration differences and concentration differences adjusted for transfer of water across the placenta are shown. Significant differences after adjustment for multiple testing by controlling the false discovery rate (FDR) according to the method of Benjamini and Hochberg are marked in bold. Note that the FDR adjusted p-values for significant results are reported in the text. (DOCX) [file pone.0185760.s001.docx]

| **Amino acid** | **Radial artery and umbilical vein** | | | **Maternal arteriovenous differences and**  **fetal venoarterial differences** | | | | **Maternal arteriovenous differences and umbilical vein** | | | | **Maternal arteriovenous differences and umbilical artery** | | | | **Radial artery and**  **fetal venoarterial differences** | | | |
| --- | --- | --- | --- | --- | --- | --- | --- | --- | --- | --- | --- | --- | --- | --- | --- | --- | --- | --- | --- |
| N | 166 | | | 147 | | | | 164 | | | | 149 | | | | 149 | | | |
|  |  | | **Crude** | | | **Adjusted for transfer of water across the placenta** | | **Crude** | | **Adjusted for transfer of water across the placenta** | | **Crude** | | **Adjusted for transfer of water across the placenta** | | **Crude** | | **Adjusted for transfer of water across the placenta** | |
| **Essential** | **r_s_** | **p-value** | **r_s_** | | **p-value** | **r_s_** | **p-value** | **r_s_** | **p-value** | **r_s_** | **p-value** | **r_s_** | **p-value** | **r_s_** | **p-value** | **r_s_** | **p-value** | **r_s_** | **p-value** |
| Histidine | **0.53** | <0.001 | -0.05 | | 0.95 | 0.000 | 0.99 | -0.08 | 0.32 | -0.06 | 0.44 | -0.08 | 0.34 | -0.06 | 0.45 | 0.15 | 0.07 | 0.19 | 0.02 |
| Isoleucine | **0.45** | <0.001 | 0.000 | | 0.97 | 0.002 | 0.98 | 0.002 | 0.98 | 0.009 | 0.91 | 0.06 | 0.46 | 0.07 | 0.37 | 0.08 | 0.34 | 0.09 | 0.26 |
| Leucine | **0.79** | <0.001 | 0.03 | | 0.71 | 0.03 | 0.71 | -0.02 | 0.85 | 0.006 | 0.94 | -0.08 | 0.34 | -0.06 | 0.47 | **0.31** | <0.001 | **0.37** | <0.001 |
| Lysine | **0.38** | <0.001 | 0.006 | | 0.94 | 0.006 | 0.94 | 0.000 | 0.99 | 0.005 | 0.95 | 0.07 | 0.43 | 0.07 | 0.41 | 0.02 | 0.86 | 0.03 | 0.72 |
| Methionine | **0.24** | 0.002 | 0.08 | | 0.34 | 0.08 | 0.36 | -0.02 | 0.77 | -0.02 | 0.79 | -0.06 | 0.44 | -0.06 | 0.47 | 0.09 | 0.27 | 0.10 | 0.21 |
| Phenylalanine | **0.28** | <0.001 | 0.18 | | 0.04 | 0.16 | 0.06 | -0.09 | 0.25 | -0.09 | 0.28 | **-0.22** | 0.006 | -0.22 | 0.007 | 0.12 | 0.16 | 0.12 | 0.13 |
| Threonine | **0.42** | <0.001 | -0.002 | | 0.98 | 0.001 | 0.99 | -0.03 | 0.68 | -0.02 | 0.79 | 0.001 | 0.99 | 0.01 | 0.88 | -0.04 | 0.66 | -0.01 | 0.88 |
| Tryptophan | 0.14 | 0.07 | 0.11 | | 0.17 | 0.10 | 0.22 | -0.05 | 0.49 | -0.05 | 0.51 | -0.13 | 0.12 | -0.12 | 0.13 | -0.09 | 0.26 | -0.08 | 0.32 |
| Valine | **0.62** | <0.001 | -0.04 | | 0.60 | -0.05 | 0.56 | -0.13 | 0.11 | -0.11 | 0.18 | -0.15 | 0.07 | -0.13 | 0.11 | 0.14 | 0.08 | 0.18 | 0.03 |
| **Non-essential** |  |  |  | |  |  |  |  |  |  |  |  |  |  |  |  |  |  |  |
| Alanine | **0.52** | <0.001 | 0.14 | | 0.10 | 0.14 | 0.09 | 0.06 | 0.44 | 0.07 | 0.35 | 0.004 | 0.96 | 0.008 | 0.92 | **0.27** | 0.001 | **0.29** | 0.001 |
| Arginine | **0.35** | <0.001 | 0.07 | | 0.40 | 0.07 | 0.41 | -0.09 | 0.24 | -0.08 | 0.30 | -0.09 | 0.29 | -0.08 | 0.32 | 0.09 | 0.28 | 0.12 | 0.15 |
| Aspartate | **0.67** | <0.001 | 0.05 | | 0.53 | 0.06 | 0.46 | 0.03 | 0.73 | 0.04 | 0.65 | -0.04 | 0.63 | -0.03 | 0.68 | 0.13 | 0.11 | 0.16 | 0.05 |
| Cysteine | **0.46** | <0.001 | -0.04 | | 0.61 | -0.04 | 0.62 | -0.2 | 0.85 | -0.003 | 0.97 | 0.3 | 0.76 | 0.04 | 0.65 | -0.001 | 0.99 | 0.03 | 0.76 |
| Glutamic acid | **-0.20** | 0.01 | 0.05 | | 0.52 | 0.05 | 0.57 | -0.12 | 0.12 | -0.12 | 0.12 | -0.17 | 0.04 | -0.17 | 0.04 | -0.10 | 0.90 | -0.003 | 0.97 |
| Glutamine | **0.58** | <0.001 | 0.01 | | 0.89 | 0.01 | 0.87 | 0.03 | 0.67 | 0.05 | 0.53 | 0.06 | 0.48 | 0.07 | 0.39 | -0.1 | 0.91 | 0.03 | 0.71 |
| Glycine | **0.31** | <0.001 | 0.13 | | 0.12 | 0.12 | 0.14 | -0.02 | 0.84 | -0.01 | 0.86 | **-0.21** | 0.009 | -0.21 | 0.01 | 0.13 | 0.11 | 0.14 | 0.09 |
| Proline | **0.42** | <0.001 | -0.01 | | 0.90 | -0.02 | 0.83 | -0.13 | 0.11 | -0.11 | 0.15 | -0.12 | 0.14 | - 0.11 | 0.18 | -0.09 | 0.26 | -0.07 | 0.40 |
| Serine | **0.33** | <0.001 | 0.03 | | 0.73 | 0.03 | 0.74 | -0.08 | 0.34 | -0.07 | 0.38 | -0.10 | 0.24 | -0.09 | 0.28 | -0.02 | 0.80 | -0.003 | 0.97 |
| Tyrosine | **0.51** | <0.001 | 0.15 | | 0.07 | 0.15 | 0.64 | 0.09 | 0.24 | 0.10 | 0.20 | -0.04 | 0.62 | -0.03 | 0.68 | 0.03 | 0.75 | 0.06 | 0.49 |
